# The Otago Exercise Program With or Without Motivational Interviewing for Community-Dwelling Older Adults: A 12-Month Follow-Up of a Randomized, Controlled Trial

**DOI:** 10.1177/0733464820902652

**Published:** 2020-03-02

**Authors:** Susanna Tuvemo Johnson, Elisabeth Anens, Ann-Christin Johansson, Karin Hellström

**Affiliations:** 1 Uppsala University, Sweden; 2 Mälardalen University, Västerås, Sweden

**Keywords:** falls, physical function, exercise, health behaviors, Otago Exercise Program, motivational interviewing, older adults

## Abstract

The aim of this randomized controlled trial was to examine the 12-month effects of the home-based Otago Exercise Program (OEP) with or without the support of motivational interviewing (MI) on community-dwelling people 75 years and older who needed walking aids and/or home help service. In total, 175 participants were randomized into three groups: OEP (*n* = 61), OEP with MI (*n* = 58), and a control group (*n* = 56) (*M* age = 83 years). Measures were physical performance, physical activity level, balance, grip strength, fall-related self-efficacy, fall rate, and fall injury rate. The OEP with and the OEP without MI, with the support of a physical therapist (six home visits and three phone calls), demonstrated no benefits in any of the measures compared with a control group. In this subgroup of pre-frail older adults, more frequent support by personnel may be required to secure efficient intensity and progression in the exercises performed on your own at home.

## Introduction

The risk factors contributing to falls and fall-related injuries among community-dwelling older adults have been studied to a great extent, and intrinsic, extrinsic, and behavioral factors have been identified (Boelens et al., 2013; Deandrea et al., 2010). Lower limb weakness (Moreland et al., 2004) and balance and gait limitations (Ambrose et al., 2013) are among the strongest modifiable intrinsic risk factors for falls. Exercise programs designed to enhance physical function and to support fall prevention in older adults have been investigated in a substantial number of studies over the last few decades, and the scientific evidence for exercise types and specified minimum interventions for effective fall preventive actions have been summarized in systematic reviews (Gillespie et al., 2012; Sherrington et al., 2008). However, the challenge of determining the best exercise program design for subgroups of individuals remains. One program that meets the suggested criteria of specified dosage (a minimum of 2 times per week) and exercise types (strength, balance, and endurance training) (Sherrington et al., 2008) is the Otago Exercise Program (OEP; Gardner et al., 2001). The program is an individually tailored, progressive, and well-structured exercise program that is suitable for performance at home (Campbell et al., 1997). The OEP has been evaluated in numerous studies in different countries and settings (Benavent-Caballer et al., 2016; Day, 2011; Shubert et al., 2018; Yoo et al., 2013). To the best of our knowledge, the OEP has not been evaluated with long-term follow-up in a Swedish context.

Although evidence for *what* to do to prevent falls in older people is becoming evident (Gillespie et al., 2012), determining *how* to motivate older persons to perform and maintain exercise programs remains a challenge. Adherence to exercise programs is generally low in older adults and decreases with age (Picorelli et al., 2014). Motivational interviewing (MI) is a communication method that is commonly used in health care settings (Rollnick et al., 2008). The use of MI as a tool to enhance physical activity levels in older adults in primary care has been evaluated with promising results, but this approach must be further investigated (Purath et al., 2014). A study on adherence to exercise over 1 year displayed that activity habits at baseline and exercising in combination with MI had significant association to exercise adherence (Arkkukangas et al., 2018). A 3-month follow-up of a randomized controlled trial (RCT) of home-based OEP and OEP + MI displayed interesting results regarding physical performance, fall self-efficacy, and grip strength in community-dwelling older adults who need walking aids and/or home support (Arkkukangas et al., 2019). The follow-up in that study was short, and also long-term perspectives must be evaluated. The aim of this study was to examine the 12-month effects of a home-based OEP, according to the original OEP protocol, with or without MI as compared with a control group on physical performance, walking speed, grip strength, physical activity level, balance, fall self-efficacy, fall rate, and fall injury rate in community-dwelling older adults who needed walking aids and/or home help service.

## Method

This three-center study was a three-armed RCT with two intervention groups and one control group. At the beginning of the RCT, a feasibility study conducted with 45 older adults and 12 physical therapists (PTs) confirmed that the study protocol had acceptable feasibility (Arkkukangas et al., 2015). The Regional Ethics Review Board of Uppsala, Sweden, approved the study protocol (Dnr 2012/147), and the study was registered at ClinicalTrials.gov (No. NCT01778972). The study followed the Declaration of Helsinki, which concerns informed consent, human rights, and correct procedures regarding treatment in research involving human participants. The participants received no compensation for their participation.

### Participants and Recruitment Procedure

**Figure 1.**
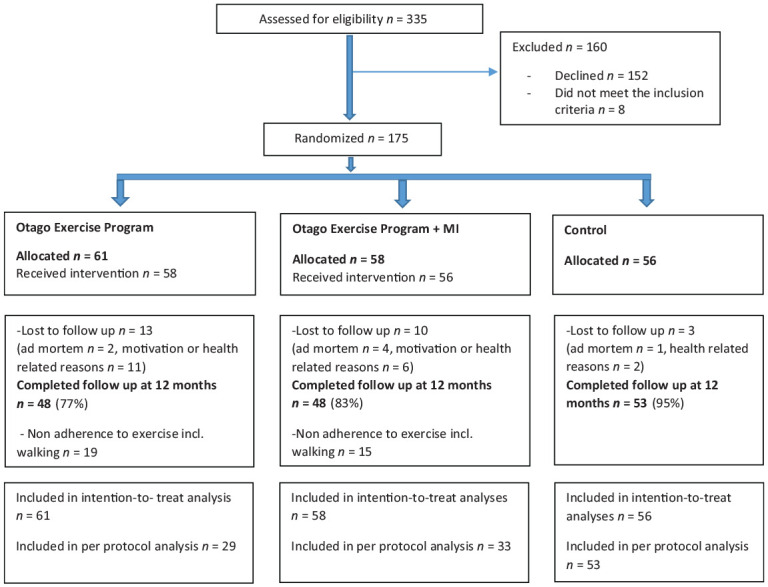
CONSORT flowchart of the participants. *Note.* MI = motivational interviewing; CONSORT = the consolidated standards of reporting trials

This study is a long-term follow-up on the same sample that was studied in a 3-month follow-up on fall preventive exercise with or without behavioral support (Arkkukangas et al., 2019). The participants were recruited by PTs or occupational therapists when walking aids were prescribed or by care managers when the older adults applied for home help service. The inclusion criteria for the RCT were the following: an age of 75 years and older, living in the community, ability to understand oral and written information in the Swedish language, and ability to walk independently indoors with or without assistive devices. Individuals with scores less than 25/30 on the Mini-Mental State Examination (MMSE; Folstein et al., 1975), ongoing physical therapy treatment due to illness or injury, or receiving terminal care were excluded. In total, 335 older adults in three Swedish municipalities were assessed for eligibility, and 175 individuals met the inclusion criteria and were willing to participate, 122 women and 53 men, *M* age = 83.2 (4.6) years (Figure 1). All of the participants agreed to their participation orally and signed a written consent form prior to enrollment in the study. The participants were randomly allocated to one of three groups as follows: the OEP, the OEP combined with MI, or the control group. The recruitment was conducted from October 2012 to May 2015.

### Measurements

The measurements and physical tests were performed in a single-blinded manner in the participants’ homes or at a health care center. Measurements and testing were completed by six independent and experienced PTs (assessors) at baseline, 3 months (Arkkukangas et al., 2019), and 12 months. The assessors were blinded to the group assignment. The assessors met in half-day meetings prior to the start of the study (2 times) and during the study process (2 times) to practice and discuss the details regarding the test and measurement manuals to strengthen interrater reliability across settings.

At baseline, a questionnaire was completed to capture a thorough understanding of the participants’ background characteristics. The questionnaire included self-reported fall frequencies and fall injuries over the last previous year, home help utilization, medication, housing and living arrangements, use of walking aids, and self-rated health-related quality of life (EuroQol 5 Dimensions Visual Analogue Scale; Greiner et al., 2003). The Mini-Nutritional Assessment (MNA)–short form was used to assess nutritional status (Guigoz, 2006), and the Geriatric Depression Scale–20 (GDS-20) assessed mental health (Gottfries et al., 1997).

Multiple modes of assessments were utilized to capture a thorough understanding of the subjects’ capacities over 1 year. The evaluated variables at baseline and at 12 months were as follows.

The Short Physical Performance Battery (SPPB; Guralnik et al., 1994) was used to assess the physical performance of the lower limbs. The test contains three components (static balance, self-selected walking speed, and sit to stand). The scale scores range from 0 to 4 points in each of the three components. A total of 12 points represents the best performance. The test has been reported to have good test–retest reliability, to be predictive of disability (Guralnik et al., 1994), and to be sensitive to changes (Ostir et al., 2002). Low scores (≤6) on the SPPB are associated with a higher fall rate in old people of both genders (Veronese et al., 2014). A clinically meaningful change (CMC) is defined as 1.0 point for SPPB (Bean et al., 2010).

Walking speed was measured from the time taken to walk a 3.0-meter-long path at a self-selected walking speed. Self-selected walking speed is associated with survival (Studenski et al., 2011), risk of future falls (Middleton, Fulk, Herter, et al., 2016), and daily ambulatory activity in older adults (Middleton, Fulk, Beets, et al., 2016). A CMC for walking speed is defined as 0.1 m/s (Bean et al., 2010).

Hand-grip muscle strength was measured using the Jamar Hand Dynamometer. The instrument has an excellent test–retest reliability for grip strength measurement (Savva et al., 2014) and provides a valid measure of general body strength (Mathiowetz et al., 1984). Grip strength predicts physical functioning (Bohannon, 2015), and impaired physical functioning indicates risk for falls (Deandrea et al., 2010). According to Kim et al. (2014), a CMC is defined as 6.5 kg.

Balance was measured using the Mini-Balance Evaluation Systems Test (Mini-BESTest; Franchignoni et al., 2010). The test consists of 14 different tasks with a scale score in each task ranging from 0 (worst performance) to 2 (best performance) for a maximum score of 28 points. The Mini-BESTest has been reported to have high interrater reliability and test–retest reliability (Tsang et al., 2013). A minimal clinical important difference (MCID) in Mini-BESTest is defined as 4 points (Godi et al., 2013).

The Frändin–Grimby Activity Scale was used to measure the level of physical activity (Frändin & Grimby, 1994). The activity level is estimated on a scale from 1 to 6 points, with 1 point representing “hardly active at all,” and 6 points representing “intensive exercise regularly and several times per week.” The Frändin–Grimby Activity Scale has been shown to have good validity (Frändin & Grimby, 1994).

The Falls Efficacy Scale–Swedish version (FES(S); K. Hellström et al., 2003) was used to measure self-confidence in an individual’s ability to perform various daily activities without falling. The instrument consists of 13 items. Each item is rated from 0 to 10 points for a maximum score of 130 points, which represents the highest level of self-efficacy. The instrument consists of six items related to personal activities of daily living (ADLs) and six items related to instrumental ADL, and one item is related to stair walking. Fall-related self-efficacy in instrumental ADL is associated with falls in community-dwelling older adults (.K. Hellström et al., 2013). The test–retest reliability for the FES(S) has been reported to be high (K. Hellström & Lindmark, 1999).

The data collection for fall events was conducted via daily reports from the participants in calendars for falls. These calendars were mailed monthly for 12 months to the research teams in return envelopes (Lamb et al., 2005). A fall was defined as “an event in which a person unintentionally comes to rest on the floor or ground, regardless of the cause and the consequences of the fall” (Jensen et al., 2002, p. 734). All falls were followed up monthly via standardized telephone interviews performed by research team members.

Exercise adherence was monitored in the two intervention groups via daily reports in exercise diaries. These diaries were mailed to the research teams, monthly for 12 months. If a fall calendar or an exercise diary was not returned on time, the participant was reminded via a telephone call. The self-documented home-exercise diary has been found to be an acceptable and valid method to reflect home-based exercise participation (Lahham et al., 2018).

### Randomization

A statistician independent from the research group generated the random allocation, and the sequences were transferred to consecutively numbered envelopes. The participants were randomly assigned to one of three groups, OEP, OEP + MI, or control, via block randomization (three, six, nine, and 12 participants in each block), which was performed by two researchers who did not participate in the intervention or data collection. The randomization was stratified by the three municipalities.

### Interventions

In conjunction with the baseline assessment, the participants in all three randomization groups received a booklet with general safety recommendations for older adults.

#### The OEP group

Within a week from randomization, the participants in the OEP group received a home visit from an experienced PT who introduced a home-based exercise program, the OEP. The participants received an exercise manual with pictures and description of each exercise, and an individually tried out ankle weight cuff. The exercise program included balance training, strength training, and walks, and was individually adjusted and increased to secure the safety and level of intensity. Each home-exercise session was estimated to take 30 min and to be performed at a rate of 3 times per week. The program included balance exercises in standing, walking in different directions, and rising from a chair. The antigravity muscle-strength exercises included knee extension (seated) and flexion (standing) and hip abduction and extension (standing) with or without ankle weight cuffs, and ankle plantar and dorsiflexion (standing). Walks were endorsed on days when no exercise was performed for a minimum of 3 times per week (Campbell et al., 1997). In total, the experienced PT made six home visits and three phone calls over 12 months to support and adjust the exercise performances. Each home visit lasted approximately 1 hr.

#### The OEP + MI group

The OEP+ MI group received the OEP (described above) in combination with MI, at all the visits and phone calls. The home visits in the OEP + MI group started with a collaborative conversation with open-ended questions, affirmations, reflective listening, and summaries (OARS) according to the MI to reinforce and activate the participants’ intrinsic resources (Miller & Rollnick, 2002). The home visit proceeded with discussion and work out on an individually tailored exercise program. The aim of the MI was to facilitate the participants’ individual decisions about the exercise plan and to enhance the persons’ own motivation and maintenance regarding the OEP training. Both the structure of the OEP and the principles of the MI were present during the six home visits and the three phone calls. Each of the home visits lasted approximately 1 hr.

#### Treatment fidelity

The structure and procedure of the original OEP protocol (Campbell et al., 1997) were introduced to each of the PTs who were involved in the interventions at meetings prior to intervention start. The exercise program protocol which included the exercise plan and the adjustments for each participant was filled out by the PTs. Each of the 11 PTs was linked to either the OEP group or the OEP + MI group to secure no diffusion of treatment. The PTs who performed the OEP + MI intervention were all experienced and acquainted with the MI method. In addition, they received a 3-day MI education session and three booster sessions by two MINT (Motivational Interviewing Network of Trainers) instructors. The education sessions contained MI theory, MI conversation practice, and feedback from instructors. Control coding of the MI conversations (performed by the Motivational Interviewing Coding Laboratory [MIC lab] in Sweden) showed an acceptable score of 3.8 on a 5-point scale, with 5 points representing high MI spirit (Jelsma et al., 2015). To further strengthen treatment fidelity, meetings were held before the start of the study and during the study period for the PTs who delivered the OEP (three meetings) and the OEP + MI (five meetings) to reach a consensus regarding program delivery and administration. During the home visits, the exercise frequency and intensity were registered according to the study protocol.

### Data Analysis

The sample size was calculated before the study start and was based on the SPPB. With a power of 80%, an alpha level of 5%, a drop-out rate estimated at 15%, and a standard deviation (*SD*) of 1.5 in the SPPB (Bean et al., 2010), a minimum of 45 participants were needed in each group. The statistical analysis was performed based on an intention-to-treat (ITT) analysis. The last value carried forward (LVCF) was used for imputation. As recommended in the literature (Ranganathan et al., 2016), a complementary per protocol (PP) analysis was performed. In the PP analysis, adherence to the protocol was set at a minimum of two walks per week plus two exercise sessions per week, which is a commonly used and accepted dosage for exercise prescription in this age group (Gardner et al., 2001). Differences between groups were analyzed with the Kruskal–Wallis independent-samples test and one-way analysis of variance (ANOVA). Within-group differences were analyzed with the paired *t* test and Wilcoxon’s signed-rank test. The significance level was set at *p* ≤ .05, and the analyses were performed using the IBM SPSS statistics software, Version 24 (SPSS Inc., Chicago, IL, USA). Falls and fall-related injuries are presented as rates/person (ITT analysis) and rates/person/year (±30 days; PP analysis), and as numbers. Injuries are presented according to their severity as minor injuries (no professional assessment required), moderate injuries (medical/health professional examination), and serious injuries (emergency or inpatient treatment) (Schwenk et al., 2012).

## Results

A total of 175 participants were enrolled. No significant baseline differences were observed among the three groups, the OEP (*n* = 61), OEP + MI (*n* = 58), and control (*n* = 56), in any of the measures presented in Table 1. Of the participants, 48 individuals (79%) in the OEP group, 48 individuals (83%) in the OEP + MI group, and 53 individuals (95%) in the control group completed the follow-up at 12 months (Figure 1). No significant differences were found in any of the baseline measures between those who were lost to follow-up and those who completed the 12-month follow-up. Of the remaining 96 participants in the two intervention groups, 62 individuals (65%) met the PP criteria (OEP, *n* = 29 and OEP + MI, *n* = 33) of 208 exercise sessions including walks (two walks plus two exercise sessions per week) over the 12-month period. Three home-exercise sessions per week were achieved by 13 participants in the OEP group (21% of allocated) and by 13 participants in the OEP + MI group (22% of allocated), and a frequency of three exercise sessions + three walks per week were achieved by nine participants in each of the intervention groups. Further information regarding adherence data related to this study has been analyzed and presented earlier (Arkkukangas et al., 2018).

**Table 1.** Baseline Characteristics and Baseline Test Outcomes of the Study Participants: *n* = 175, Numbers and (%), Outcomes, *M (SD)*/*median [IQR]*.

Characteristics	OEP (*n* = 61)	OEP + MI (*n* = 58)	Control (*n* = 56)	Total (*N* = 175)
Age, years	*83.4 (5.0)*	*83.7 (4.1)*	*82.3 (4.7)*	*83 (4.7)*
Women/men	41/20 (67.2/32.8)	40/18 (69.0/31.0)	41/15 (73.2/26.8)	122/53 (69.7/30.3)
Highest level of education				
Elementary or junior secondary high/girls’ school	37 (60.7)	29 (50.0)	35 (62.5)	101 (57.7)
Upper secondary school or university/college	24 (39.3)	29 (50.0)	21 (37.5)	74 (42.3)
Housing and living arrangement				
Apartment block	47 (77.0)	43 (74.1)	45 (79.4)	135 (77.1)
Townhouse or villa	14 (23.0)	15 (25.9)	11 (19.6)	40 (22.8)
Living alone/living with someone	34/27 (55.7/42.3)	33/25 (56.9/43.1)	36/20 (64.3/35.7)	103/72 (58.9/41.1)
Assistance with daily activities				
No	35 (57.4)	33 (56.9)	30 (53.6)	98 (56.0)
Yes, from relatives	14 (23.0)	10 (17.2)	10 (17.9)	34 (19.4)
Yes, from home assistance	12 (19.7)	15 (25.9)	16 (28.6)	43 (24.6)
Walking aid				
No	5 (8.2)	6 (10.3)	3 (5.4)	14 (8.0)
Yes, outdoors	46 (75.4)	41 (70.7)	45 (80.4)	132 (75.4)
Yes, indoors	10 (16.4)	11 (19.0)	8 (14.3)	29 (16.6)
Hours/week, moderate physical activity, *n* = 173				
≤3 hr per week	46 (76.7)	43 (74.1)	39 (71.0)	128 (74.0)
˃3 hr per week	14 (23.3)	15 (25.9)	16 (29.0)	45 (26.0)
Fallen last 12 months before baseline, *n* = 174				
No	37 (60.7)	30 (51.7)	34 (61.9)	101 (58.0)
Yes, one fall	13 (21.3)	14 (24.1)	11 (20.0)	38 (21.8)
Yes, two or more falls	11 (18.0)	14 (24.1)	10 (18.2)	35 (20.1)
Disorders				
Heart and coronary	37 (60.1)	22 (38.0)	29 (57.8)	88 (50.3)
Urinary	10 (16.4)	13 (22.4)	13 (23.2)	36 (20.6)
Visual^a^	5 (8.2)	11 (19.0)	12 (21.4)	28 (16.0)
Neurological	5 (8.2)	2 (3.5)	4 (7.1)	11 (6.3)
Medication, *n* = 173				
Blood pressure medicine	45 (75.0)	36 (63.2)	43 (76.8)	124 (71.7)
Diuretic	24 (40.0)	12 (21.1)	18 (32.1)	54 (31.2)
Hypnotics	17 (28.3)	20 (35.1)	20 (35.7)	57 (32.9)
Tranquilizer	3 (5.8)	7 (12.3)	3 (5.4)	13 (7.5)
Antidepressants	6 (10.0)	4 (7.0)	4 (7.1)	14 (7.7)
Test outcomes				
SPPB (0–12 points)	*7.9 (2.4)*	*7.8 (2.5)*	*7.7 (2.4)*	*7.7 (2.4)*
Walking speed (m/s)	*0.69 (0.2)*	*0.70 (0.2)*	*0.66 (0.2)*	*0.69 (0.2)*
Grip strength right hand (kg)	*25.9 (8.5)*	*24.2 (7.7)*	*24.4 (7.3)*	*24.5 (7.8)*
Mini-BESTest (0–28 points)	*16 [6]*	*16 [8]*	*16 [7]*	*16 [7]*
Frändin–Grimby (0–6 points)	*3 [1]*	*3 [1]*	*3 [0]*	*3 [1]*
FES(S) (0–130 points)	*103 [31]*	*107.5 [35]*	*104.4 [41]*	*107 [33]*
Mini-Mental State Examination (0–30 points)	*28 [3]*	*28 [2]*	*28 [2]*	*28 [2]*
Mini-Nutritional Assessment (0–14 points)	*13 [2]*	*13 [2]*	*13 [3]*	*13 [2]*
Geriatric Depression Scale (0–20 points)	*4 [3]*	*4 [3]*	*5 [3]*	*4 [3]*
EQ-5D VAS (0–100)	*64.6 (14.5)*	*62.1 (16.3)*	*60.7 (15.5)*	*63.9 (15.5)*

*Note. SD* = standard deviation; IQR = interquartile range; OEP = Otago Exercise Program; MI = motivational interviewing; SPPB = Short Physical Performance Battery; Mini-BESTest = Mini-Balance Evaluation Systems Test; FES(S) = Falls Efficacy Scale–Swedish version; EQ-5D VAS = EuroQol 5 Dimensions Visual Analogue Scale.

a Not correctable by glasses.

### Effects Between Groups and Within Groups

The between-group data analyses showed no significant differences among the three groups in the scores of the SPPB, the walking speed, or the grip strength (Table 2). Nor did between-group analysis display significant differences in the Mini-BESTest scores, the Frändin–Grimby Activity Scale, or the FES(S) scores (Table 2).

**Table 2.** Outcome Measures in Mean or *Median* Changes From Baseline to the 12-Month Follow-Up: *p* Values for the **Between Groups** Analyses Based on ITT and PP.

Outcomes	Otago group	Otago + MI group	Control group	*p* value
	*M (SD)*/*Mdn (IQR)* change	*M (SD)*/*Mdn (IQR)* change	*M (SD)*/*Mdn (IQR)* change	
Analysis of ITT	*n* = 61	*n* = 58	*n* = 56	
SPPB (0–12 points)	−0.05 (1.8)	−0.10 (2.2)	0.12 (1.9)	.81
Walking speed (m/s)	−0.02 (0.2)	−0.02 (0.2)	−0.01 (0.1)	.99
Grip strength, right hand (kg)	0.58 (3.6)	−0.54 (4.5)	−0.05 (4.1)	.32
Mini-BESTest (0–28 points)	*0.0 (5.0)*	*0.0 (5.0)*	*−0.5 (4.8)*	.91
Frändin–Grimby (0–6 points)	*0.0 (0.0)*	*0.0 (0.2)*	*0.0 (1.0)*	.16
FES(S) (0–130 points)	*0.0 (26.5)*	*0.0 (24.5)*	*−3.0 (21.5)*	.58
Analysis PP	*n* = 28–29	*n* = 27–33	*n* = 52–53	
SPPB (0–12 points)	0.5 (2.0)	0.0 (2.4)	0.3 (2.4)	.71
Walking speed (m/s)	0.03 (0.2)	−0.03 (0.2)	−0.01 (0.2)	.45
Grip strength, right hand	0.5 (4.6)	−0.6 (5.3)	−0.3 (4.2)	.65
Mini-BESTest (0–28 points)	*0.0 (6.0)*	*0.0 (8.0)*	*−1.0 (4.8)*	.58
Frändin–Grimby (1–6 points)	*0.0 (1.0)*	*0.0 (1.0)*	*0.0 (1.0)*	.41
FES(S) (0–130 points)	*0.0 (33.0)*	*0.0 (33.0)*	*−3.0 (22.5)*	.83

*Note.* ITT = intention to treat; PP = per protocol; MI = motivational interviewing; *SD* = standard deviation; IQR = interquartile range; SPPB = Short Physical Performance Battery; Mini-BESTest = Mini-Balance Evaluation Systems Test; FES(S) = Falls Efficacy Scale, Swedish version.

*p* ≤ .05.

No significant within-group differences were seen among the three groups in any of the measures: the SPPB, the walking speed, the grip strength, the Mini-BESTest, the Frändin–Grimby, and the FES(S) (Table 3). CMCs or MCIDs per group in four of the variables are displayed in Table 3.

**Table 3.** Outcome Measures in Mean or *Median* Outcomes at Baseline and 12 Months: **Within Group**, Analysis PP, and *p* Values for Differences.

Outcomes Analysis PP	Baseline Mean or *median* (minimum–maximum)	12 Months Mean or *median* (minimum–maximum)	*p* value	CMC/MCID improvement *n* (%)
SPPB (0–12 points)				
Otago (*n* = 29)	8.1 (4–12)	8.7 (2–12)	.2	13 (45)
Otago + MI (*n* = 33)	7.4 (1–12)	8.1 (2–12)	.9	14 (42)
Control (*n* = 52)	7.7 (3–12)	8.0 (1–12)	.6	24 (46)
Walking speed (m/s)				
Otago (*n* = 28)	0.72 (0.46–1.30)	0.74 (0.35–1.23)	.5	9 (32)
Otago + MI (*n* = 28)	0.73 (0.21–1.34)	0.70 (0.36–1.23)	.3	7 (25)
Control (*n* = 52)	0.68 (0.37–1.07)	0.67 (0.34–1.00)	.7	13 (25)
Grip strength, right hand (kg)				
Otago (*n* = 29)	23.8 (10–40)	24.3 (12–36)	.7	3 (10)
Otago + MI (*n* = 33)	25.0 (10–44)	24.4 (8–40)	.6	3 (9)
Control (*n* = 53)	24.4 (12–48)	24.3 (12–46)	.4	2 (4)
Mini-BESTest (0–28 points)				
Otago (*n* = 28)	*16* (8–27)	*16* (5–26)	.9	5 (18)
Otago + MI (*n* = 27)	*19* (3–26)	*16* (2–26)	.4	2 (7)
Control (*n* = 52)	*16.5* (4–28)	*16.5* (1–24)	.1	8 (15)
Frändin–Grimby (0–6 points)				
Otago (*n* = 29)	*3.0* (1–5)	*3.0* (1–4)	.6	
Otago + MI (*n* = 29)	*3.0* (1–4)	*3.0* (2–4)	.1	
Control (*n* = 53)	*3.0* (1–5)	*3.0* (1–5)	.1	
FES(S) (0–130 points)				
Otago (*n* = 28)	*108* (62–130)	*107* (43–130)	.5	
Otago + MI (*n* = 28)	*109* (60–130)	*107.5* (48–130)	.5	
Control (*n* = 53)	*105* (44–130)	*101* (40–130)	.1	

*Note.* CMC improvements of 1 point (SPPB) and 0.1 m/s (gait speed); hand grip 6.5 kg; MCID improvements of 4 points (Mini-BESTest). PP = per protocol; CMC = clinically meaningful change; MCID = minimal clinical important difference; SPPB = Short Physical Performance Battery; MI = motivational interviewing; Mini-BESTest = Mini-Balance Evaluation Systems Test; FES(S) = Falls Efficacy Scale–Swedish version.

### Falls and Fall Injuries

In total, 74 individuals (42%) reported one or more falls, of which 53 participants (30%) were injured (injury rate 0.47). Seven injuries were fractures. Overall, 185 falls occurred (fall rate 1.06) over the 12-month period. One fall occurred during the exercise (in the OEP + MI group); this fall did not result in an injury. Fall rates in the three groups were 1.1 in the OEP group, 1.4 in the OEP + MI group, and 0.6 in the control group. The differences were not significant (Table 4). Injury rate did not differ significantly between the groups. Most injuries were of the minor type in all groups.

**Table 4.** Fall Rate, Number of Fallers, Injury Rate, and Fall Injuries in 12 Months, Analyses Based on ITT and PP, *p* Values for Differences **Between Groups**.

ITT				
Outcomes	OEP *n* = 61	OEP + MI *n* = 58	Control *n* = 56	*p* value
Falls				
Fall rate/person^a^	1.1	1.4	0.6	.37
Fallers, *n* (%)	22 (36)	33 (57)	19 (34)	
One fall, *n* (%)	13 (21)	18 (31)	9 (16)	
Two to four falls, *n* (%)	7 (11)	11 (26)	8 (14)	
Five or more falls, *n* (%)	2 (3)	4 (7)	2 (4)	
Number of falls	70	79	36	
Fall injuries				
Injury rate/person^a^	0.5	0.6	0.3	.36
Injured participants, *n* (%)	16 (25)	25 (41)	12 (21)	
Number of injuries (% of falls)	28 (49)	36 (46)	19 (53)	
Minor (% of falls)	*20 (29)*	*30 (38)*	*10 (28)*	
Moderate (% of falls)	*7 (10)*	*3 (4)*	*6 (17)*	
Serious (% of falls)	*1 (1)*	*3 (4)*	*3 (8)*	
PP				
	*n* = 29	*n* = 31	*n* = 53	
Falls				
Fall rate/person/year	1.0	1.1	0.7	.28
Fallers, *n* (%)	14 (48)	15 (48)	18 (34)	
One fall, *n* (%)	8 (28)	11 (35)	9 (17)	
Two to four falls, *n* (%)	6 (21)	4 (13)	7 (13)	
Five or more falls, *n* (%)	1 (3)	1 (3)	2 (4)	
Number of falls	30	35	36	
Fall injuries				
Injury rate/person/year	0.6	0.6	0.3	.36
Injured participants, *n* (%)	10 (35)	15 (48)	11 (21)	
Number of injuries (% of falls)	18 (60)	18 (51)	14 (39)	
Minor (% of injuries)	*14 (78)*	*13 (72)*	*10 (71)*	
Moderate (% of injuries)	*4 (22)*	*4 (22)*	*2 (14)*	
Serious (% of injuries)	*0 (0)*	*1 (5)*	*2 (14)*	

*Note.* ITT = intention to treat; PP = per protocol; OEP = Otago Exercise Program; MI = motivational interviewing.

a Data regarding an exact follow-up period for every participant were not available. *p* ≤.05.

## Discussion

Our study results, based on the OEP intervention with or without MI, contrast the results of the review and meta-analysis of seven studies on the outcome of the OEP, *M* age = 81.6 (3.9) years, in New Zealand (Thomas et al., 2010). This study including Swedish participants, *M* age = 83.2 (4.7) years, did not confirm a long-term benefit of the OEP with or without MI at 1 year, compared with a control group. The Thomas et al. samples and this sample were similar in age, but otherwise, the comparability is problematic due to great differences in the selected baseline characteristic variables. A possible difference between these settings was the weather conditions. Long winters might affect the ability to walk outside. Two walks per week was achieved by 52% of participants in the intervention groups, which is less than in the Robertson et al. (2001) study, where 71% of participants reached 2 times/week of walks. However, our sample was specially selected to involve older community dwellers who needed walking aids and/or home help service, which indicated some degree of frailty and might have affected the delivered training dosage and the study results. Notably, all three groups in this sample maintained their physical function and physical activity during the assessment period; this finding can be interpreted as a promising result, because deterioration with age in muscle strength (Moreland et al., 2004), balance (Rubenstein, 2006), and physical activity (Sun et al., 2013) has been well documented. Our results indicate that attention paid to the individuals in the study and the registration of falls could have contributed to awareness of fall risk and benefits of physical activity and exercises, thereby leading to behavioral changes in all groups.

The hypothesis that the combination of OEP and MI increased physical function compared with OEP and a control group could not be confirmed. The small within-group effects reported at the 3-month follow-up on physical performance, fall-related self-efficacy, and grip strength in the OEP and OEP + MI groups (Arkkukangas et al., 2019) did not persist at 12 months. The higher number of injured individuals in the intervention groups may have been due to recall bias in the control group. The intervention groups received three visits by the assessors and an additional six visits by the PTs. These visits may have rendered the participants more observant of fall injuries, especially minor injuries, than the control group.

To attain the desired effect, an exercise program must be both safe and challenging for the individual (Sherrington et al., 2016). Only one fall occurred during an exercise session, which might indicate that the safety margin for the exercises was high in this study. The degree of progression was controlled and continuously adjusted in the prescription protocols, in conjunction with the home visits by the PTs, and the exercise session rates were followed using daily exercise diaries. As presented in a related study on the same sample, the researchers found that predictors for adherence to exercise were physical activity habits at baseline and exercise in combination with MI (Arkkukangas et al., 2018). Those results imply that MI is associated to exercise adherence. However, the level of exercise adherence in the OEP + MI group did not result in improved physical outcomes or fewer falls. The unimproved outcomes in the intervention groups might be explained by uncontrolled individual challenge in the balance and strength exercises performed without the presence of the PT. The safety margin, in combination with the uncontrolled level of the challenge, might explain some parts of the non-appearance of improvements. To our knowledge, the individual challenge in the balance and strength exercises performed on your own has not been discussed in other OEP studies. We suggest further developments of measures and instruments to control exercises performed on your own.

In an updated systematic review and meta-analysis on falls prevention by Sherrington et al. (2016), as much as 3 hr/week and a higher challenge to balance is proposed. If so, it might be a great challenge to achieve effective fall prevention via (mostly) unattended home exercises for this group of older adults. Therefore, we suggest further studies to gain increased knowledge on how to achieve clinically practicable home-based exercise programs with high enough intensity for community-dwelling older adults who need walking aids and/or home help service. In this study, the original OEP protocol was followed, with six home visits and three supportive phone calls. To support home exercises, over a period of 12 months, we suggest more frequently and regularly performed home visits and phone calls, especially for relatively frail older adults. This is supported in the literature where an alternative delivery model for the OEP recently has been explored (Shubert et al., 2017). The Shubert et al. delivery model included eight home visits and weekly phone calls over a 6-month period, *M* age = 79.8 (11.8) years.

To achieve a high level of balance challenge and high frequencies of home-exercise sessions in individuals similar to the sample in our study, we recommend further evaluations of delivery arrangements, including more frequent support from instructors. MI might be a valuable tool to support exercise adherence, but further studies are needed.

### Strengths and Limitations

High test and treatment fidelity were assured by thoroughly followed protocols in the intervention groups, regular meetings for the involved assessors and physiotherapists, and by control coding of the MI conversations. The assessors were blinded to the group allocation, and randomization was performed by researchers not included in the assessments or interventions, which could be referred to as strengths of this study. The home-exercise adherence (frequency/week) was monitored via exercise diaries, and the level of intensity (number of sets and reps, and weight on the ankle weights) was prescribed. However, the exercises were mostly performed unattended and were therefore not fully controlled. A limitation of this study refers to the possibility of generalization of the results. As all of the participants had chosen to participate in a fall prevention study, they were probably not representative of all community-dwelling older adults who need walking aids or home help service. The results should consequently be generalized with cautiousness. The higher dispersion in the SPPB variable at baseline (*SD* = 2.4) than expected (*SD* = 1.5) indicated that the study was underpowered, which was confirmed in a post hoc power analysis. A replication of the study might be worthwhile.

## Conclusion

This 12-month follow-up of the OEP with or without MI compared with a control group displayed no long-term benefits in physical performance, physical activity level, balance, grip strength, fall-related self-efficacy, fall rate, or fall injury rate compared with a control group in community-dwelling older individuals who needed walking aids and/or home help service. As the study was confirmed to be underpowered, the authors suggest replicative studies on this specific subgroup of older adults. However, to support home exercises in this subgroup, PT supervision 9 times over a 12-month period is probably not sufficient. More frequent personal support may be required to secure efficient levels of intensity and progression in exercises performed on your own at home.

## Supplemental Material

CONSORT_Checklist_190618 – Supplemental material for The Otago Exercise Program With or Without Motivational Interviewing for Community-Dwelling Older Adults: A 12-Month Follow-Up of a Randomized, Controlled Trial Click here for additional data file. Supplemental material, CONSORT_Checklist_190618 for The Otago Exercise Program With or Without Motivational Interviewing for Community-Dwelling Older Adults: A 12-Month Follow-Up of a Randomized, Controlled Trial by Susanna Tuvemo Johnson, Elisabeth Anens, Ann-Christin Johansson and Karin Hellström in Journal of Applied Gerontology

## References

[bibr1-0733464820902652] Ambrose A. F. Paul G. Hausdorff J. M. (2013). Risk factors for falls among older adults: A review of the literature. Maturitas, 75(1), 51–61. 10.1016/j.maturitas.2013.02.009 23523272

[bibr2-0733464820902652] Arkkukangas M. Johnson S. T. Hellström K. Söderlund A. Eriksson S. Johansson A.-C. (2015). A feasibility study of a randomised controlled trial comparing fall prevention using exercise with or without the support of motivational interviewing. Preventive Medicine Reports, 2, 134–140. 10.1016/j.pmedr.2015.01.007 26844061 PMC4721421

[bibr3-0733464820902652] Arkkukangas M. Soderlund A. Eriksson S. Johansson A. C. (2018). One-year adherence to the Otago Exercise Program with or without motivational interviewing in community-dwelling older adults. Journal of Aging Physical Activity, 26(3), 390–395. 10.1123/japa.2017-0009 28952864

[bibr4-0733464820902652] Arkkukangas M. Soderlund A. Eriksson S. Johansson A. C. (2019). Fall preventive exercise with or without behavior change support for community-dwelling older adults: A randomized controlled trial with short-term follow-up. Journal of Geriatric Physical Therapy, 42(1), 9–17. 10.1519/jpt.0000000000000129 28244890

[bibr5-0733464820902652] Bean J. F. Kiely D. K. LaRose S. Goldstein R. Frontera W. R. Leveille S. G. (2010). Are changes in leg power responsible for clinically meaningful improvements in mobility in older adults? Journal of the American Geriatrics Society, 58(12), 2363–2368. 10.1111/j.1532-5415.2010.03155.x 21143443 PMC3051803

[bibr6-0733464820902652] Benavent-Caballer V. Rosado-Calatayud P. Segura-Orti E. Amer-Cuenca J. J. Lison J. F. (2016). The effectiveness of a video-supported group-based Otago Exercise Programme on physical performance in community-dwelling older adults: A preliminary study. Physiotherapy, 102(3), 280–286. 10.1016/j.physio.2015.08.002 26395209

[bibr7-0733464820902652] Boelens C. Hekman E. E. G. Verkerke G. J. (2013). Risk factors for falls of older citizens. Technology and Health Care, 21(5), 521–533. 10.3233/thc-130748 24077498

[bibr8-0733464820902652] Bohannon R. W. (2015). Muscle strength: Clinical and prognostic value of hand-grip dynamometry. Current Opinion in Clinical Nutrition and Metabolic Care, 18(5), 465–470. 10.1097/mco.0000000000000202 26147527

[bibr9-0733464820902652] Campbell A. J. Robertson M. C. Gardner M. M. Norton R. N. Tilyard M. W. Buchner D. M. (1997). Randomised controlled trial of a general practice programme of home based exercise to prevent falls in elderly women. British Medical Journal, 315(7115), 1065–1069. 9366737 10.1136/bmj.315.7115.1065 PMC2127698

[bibr10-0733464820902652] Day L. (2011). The Otago strength and balance exercise programme lowers the risk of death and falls in the older people at 12 months. Evidence-Based Nursing, 14(3), 76–78. 10.1136/ebn1157 21436159

[bibr11-0733464820902652] Deandrea S. Lucenteforte E. Bravi F. Foschi R. La Vecchia C. Negri E. (2010). Risk factors for falls in community-dwelling older people: A systematic review and meta-analysis. Epidemiology, 21(5), 658–668. 10.1097/EDE.0b013e3181e89905 20585256

[bibr12-0733464820902652] Folstein S. E. Folstein M. F. McHugh P. R. (1975). “Mini-mental state”: A practical method for grading the cognitive state of patients for the clinician. Journal of Psychiatric Research, 12(3), 189–198. 10.1016/0022-3956(75)90026-6 1202204

[bibr13-0733464820902652] Franchignoni F. Horak F. Godi M. Nardone A. Giordano A. (2010). Using psychometric techniques to improve the Balance Evaluation Systems Test: The Mini-BESTest. Journal of Rehabilitation Medicine, 42(4), 323–331. 10.2340/16501977-0537 20461334 PMC3228839

[bibr14-0733464820902652] Frändin K. Grimby G. (1994). Assessment of physical activity, fitness and performance in 76-year-olds. Scandinavian Journal of Medicine & Science in Sports, 4(1), 41–46. 10.1111/j.1600-0838.1994.tb00404.x

[bibr15-0733464820902652] Gardner M. M. Buchner D. M. Robertson M. C. Campbell A. J. (2001). Practical implementation of an exercise-based falls prevention programme. Age and Ageing, 30(1), 77–83. 11322678 10.1093/ageing/30.1.77

[bibr16-0733464820902652] Gillespie L. D. Robertson M. C. Gillespie W. J. Sherrington C. Gates S. Clemson L. M. Lamb S. E. (2012). Interventions for preventing falls in older people living in the community. The Cochrane Database of Systematic Reviews, 9, Article CD007146. 10.1002/14651858.CD007146.pub3 PMC8095069 22972103

[bibr17-0733464820902652] Godi M. Franchignoni F. Caligari M. Giordano A. Turcato A. M. Nardone A. (2013). Comparison of reliability, validity, and responsiveness of the Mini-BESTest and Berg Balance Scale in patients with balance disorders. Physical Therapy, 93(2), 158–167. 10.2522/ptj.20120171 23023812

[bibr18-0733464820902652] Gottfries G. G. Noltorp S. Nørgaard N. (1997). Experience with a Swedish version of the Geriatric Depression Scale in primary care centres. International Journal of Geriatric Psychiatry, 12(10), 1029–1034. 10.1002/(SICI)1099-1166(199710)12:10<1029::AID-GPS683>3.0.CO;2-D 9395935

[bibr19-0733464820902652] Greiner W. Weijnen T. Nieuwenhuizen M. Oppe S. Badia X. Busschbach J.. . . de Charro F. (2003). A single European currency for EQ-5D health states. Results from a six-country study. The European Journal of Health Economics, 4(3), 222–231. 10.1007/s10198-003-0182-5 15609189

[bibr20-0733464820902652] Guigoz Y. (2006). The Mini Nutritional Assessment (MNA) review of the literature: What does it tell us? The Journal of Nutrition, Health & Aging, 10(6), 466–485; discussion 485–487. 17183419

[bibr21-0733464820902652] Guralnik J. M. Simonsick E. M. Ferrucci L. Glynn R. J. Berkman L. F. Blazer D. G.. . . Wallace R. B. (1994). A Short Physical Performance Battery assessing lower extremity function: Association with self-reported disability and prediction of mortality and nursing home admission. Journal of Gerontology, 49(2), M85–M94. 8126356 10.1093/geronj/49.2.m85

[bibr22-0733464820902652] Hellström K. Lindmark B. (1999). Fear of falling in patients with stroke: A reliability study. Clinical Rehabilitation, 13(6), 509–517. 10588538 10.1191/026921599677784567

[bibr23-0733464820902652] Hellström K. Lindmark B. Wahlberg B. Fugl-Meyer A. R. (2003). Self-efficacy in relation to impairments and activities of daily living disability in elderly patients with stroke: A prospective investigation. Journal of Rehabilitation Medicine, 35(5), 202–207. 14582550 10.1080/16501970310000836

[bibr24-0733464820902652] Hellström S. Sandström M. Wågert P. H. Sandborgh M. Söderlund A. Adolfsson E. T. Johansson A.-C. (2013). Fall-related self-efficacy in instrumental activities of daily living is associated with falls in older community-living people. Physical & Occupational Therapy in Geriatrics, 31(2), 128–139. 10.3109/02703181.2013.792912

[bibr25-0733464820902652] Jelsma J. G. Mertens V. C. Forsberg L. Forsberg L. (2015). How to measure motivational interviewing fidelity in randomized controlled trials: Practical recommendations. Contemporary Clinical Trials, 43, 93–99. 10.1016/j.cct.2015.05.001 25962891

[bibr26-0733464820902652] Jensen J. Lundin-Olsson L. Nyberg L. Gustafson Y. (2002). Fall and injury prevention in older people living in residential care facilities. A cluster randomized trial. Annals of Internal Medicine, 136(10), 733–741. 12020141 10.7326/0003-4819-136-10-200205210-00008

[bibr27-0733464820902652] Kim J. K. Park M. G. Shin S. J. (2014). What is the minimum clinically important difference in grip strength? Clinical Orthopaedics & Related Research, 472(8), 2536–2541. 10.1007/s11999-014-3666-y 24817380 PMC4079876

[bibr28-0733464820902652] Lahham A. McDonald C. F. Mahal A. Lee A. L. Hill C. J. Burge A. T.. . . Holland A. E. (2018). Acceptability and validity of a home exercise diary used in home-based pulmonary rehabilitation: A secondary analysis of a randomised controlled trial. The Clinical Respiratory Journal, 12(6), 2057–2064. 10.1111/crj.12773 29392881

[bibr29-0733464820902652] Lamb S. E. Jørstad-Stein E. C. Hauer K. Becker C., & Prevention of Falls Network Europe Consensus Group. (2005). Development of a common outcome data set for fall injury prevention trials: The Prevention of Falls Network Europe consensus. Journal of the American Geriatric Society, 53(9), 1618–1622. 10.1111/j.1532-5415.2005.53455.x 16137297

[bibr30-0733464820902652] Mathiowetz V. Weber K. Volland G. Kashman N. (1984). Reliability and validity of grip and pinch strength evaluations. The Journal of Hand Surgery, 9(2), 222–226. 6715829 10.1016/s0363-5023(84)80146-x

[bibr31-0733464820902652] Middleton A. Fulk G. D. Beets M. W. Herter T. M. Fritz S. L. (2016). Self-selected walking speed is predictive of daily ambulatory activity in older adults. Journal of Aging and Physical Activity, 24(2), 214–222. 10.1123/japa.2015-0104 26371593 PMC4792803

[bibr32-0733464820902652] Middleton A. Fulk G. D. Herter T. M. Beets M. W. Donley J. Fritz S. L. (2016). Self-selected and maximal walking speeds provide greater insight into fall status than walking speed reserve among community-dwelling older adults. American Journal of Physical Medicine & Rehabilitation, 95(7), 475–482. 10.1097/phm.0000000000000488 27003205 PMC4912425

[bibr33-0733464820902652] Miller W. R. Rollnick S. (2002). Motivational interviewing: Preparing people for change (2nd ed.). Guilford Press.

[bibr34-0733464820902652] Moreland J. D. Richardson J. A. Goldsmith C. H. Clase C. M. (2004). Muscle weakness and falls in older adults: A systematic review and meta-analysis. Journal of the American Geriatrics Society, 52(7), 1121–1129. 10.1111/j.1532-5415.2004.52310.x 15209650

[bibr35-0733464820902652] Ostir G. V. Volpato S. Fried L. P. Chaves P. Guralnik J. M. & Women’s Health and Aging Study. (2002). Reliability and sensitivity to change assessed for a summary measure of lower body function: Results from the Women’s Health and Aging Study. Journal of Clinical Epidemiology, 55(9), 916–921. 12393080 10.1016/s0895-4356(02)00436-5

[bibr36-0733464820902652] Picorelli A. M. A. Pereira L. S. M. Pereira D. S. Felicio D. Sherrington C. (2014). Adherence to exercise programs for older people is influenced by program characteristics and personal factors: A systematic review. Journal of Physiotherapy, 60(3), 151–156. 10.1016/j.jphys.2014.06.012 25092418

[bibr37-0733464820902652] Purath J. Keck A. Fitzgerald C. E. (2014). Motivational interviewing for older adults in primary care: A systematic review. Geriatric Nursing, 35(3), 219–224. 10.1016/j.gerinurse.2014.02.002 24656051

[bibr38-0733464820902652] Ranganathan P. Pramesh C. S. Aggarwal R. (2016). Common pitfalls in statistical analysis: Intention-to-treat versus per-protocol analysis. Perspectives in Clinical Research, 7(3), 144–146. 10.4103/2229-3485.184823 27453832 PMC4936074

[bibr39-0733464820902652] Robertson M. C. Devlin N. Gardner M. M. Campbell A. J. (2001). Effectiveness and economic evaluation of a nurse delivered home exercise programme to prevent falls. 1: Randomised controlled trial. British Medical Journal, 322(7288), 697–701. 10.1136/bmj.322.7288.697 11264206 PMC30094

[bibr40-0733464820902652] Rollnick S. Miller W. R. Butler C. C. (2008). Motivational interviewing in health care: Helping patients change behavior. Guilford Press.

[bibr41-0733464820902652] Rubenstein L. Z. (2006). Falls in older people: Epidemiology, risk factors and strategies for prevention. Age and Ageing, 35(Suppl. 2), ii37–ii41. 10.1093/ageing/afl084 16926202

[bibr42-0733464820902652] Savva C. Giakas G. Efstathiou M. Karagiannis C. (2014). Test–retest reliability of handgrip strength measurement using a hydraulic hand dynamometer in patients with cervical radiculopathy. Journal of Manipulative & Physiological Therapeutics, 37(3), 206–210. 10.1016/j.jmpt.2014.02.001 24630769

[bibr43-0733464820902652] Schwenk M. Lauenroth A. Stock C. Moreno R. R. Oster P. McHugh G.. . . Hauer K. (2012). Definitions and methods of measuring and reporting on injurious falls in randomised controlled fall prevention trials: A systematic review. BMC Medical Research Methodology, 12, Article 50. 10.1186/1471-2288-12-50 PMC3388463 22510239

[bibr44-0733464820902652] Sherrington C. Michaleff Z. A. Fairhall N. Paul S. S. Tiedemann A. Whitney J.. . . Lord S. R. (2016). Exercise to prevent falls in older adults: An updated systematic review and meta-analysis. British Journal of Sports Medicine, 51(24), 1750–1758. 10.1136/bjsports-2016-096547 27707740

[bibr45-0733464820902652] Sherrington C. Whitney J. C. Lord S. R. Herbert R. D. Cumming R. G. Close J. C. (2008). Effective exercise for the prevention of falls: A systematic review and meta-analysis. Journal of the American Geriatrics Society, 56(12), 2234–2243. 10.1111/j.1532-5415.2008.02014.x 19093923

[bibr46-0733464820902652] Shubert T. E. Goto L. S. Smith M. L. Jiang L. Rudman H. Ory M. G. (2017). The Otago Exercise Program: Innovative delivery models to maximize sustained outcomes for high risk, homebound older adults. Frontiers in Public Health, 5, Article 54. 10.3389/fpubh.2017.00054 PMC5362608 28386536

[bibr47-0733464820902652] Shubert T. E. Smith M. L. Jiang L. Ory M. G. (2018). Disseminating the Otago Exercise Program in the United States: Perceived and actual physical performance improvements from participants. Journal of Applied Gerontology, 37(1), 79–98. 10.1177/0733464816675422 27794055

[bibr48-0733464820902652] Studenski S. Perera S. Patel K. Rosano C. Faulkner K. Inzitari M.. . . Guralnik J. (2011). Gait speed and survival in older adults. The Journal of the American Medical Association, 305(1), 50–58. 10.1001/jama.2010.1923 21205966 PMC3080184

[bibr49-0733464820902652] Sun F. Norman I. J. While A. E. (2013). Physical activity in older people: A systematic review. BMC Public Health, 13, Article 449. 10.1186/1471-2458-13-449 PMC3651278 23648225

[bibr50-0733464820902652] Thomas S. Mackintosh S. Halbert J. (2010). Does the “Otago Exercise Programme” reduce mortality and falls in older adults? A systematic review and meta-analysis. Age and Ageing, 39(6), 681–687. 10.1093/ageing/afq102 20817938

[bibr51-0733464820902652] Tsang C. S. L. Liao L. R. Chung R. C. K. Pang M. Y. C. (2013). Psychometric properties of the Mini-Balance Evaluation Systems Test (Mini-BESTest) in community-dwelling individuals with chronic stroke. Physical Therapy, 93(8), 1102–1115. 10.2522/ptj.20120454 23559522

[bibr52-0733464820902652] Veronese N. Bolzetta F. Toffanello E. D. Zambon S. De Rui M. Perissinotto E.. . . Manzato E. (2014). Association between Short Physical Performance Battery and falls in older people: The Progetto Veneto Anziani Study. Rejuvenation Research, 17(3), 276–284. 10.1089/rej.2013.1491 24387140 PMC4062103

[bibr53-0733464820902652] Yoo H. N. Chung E. Lee B. H. (2013). The effects of augmented reality-based Otago exercise on balance, gait, and falls efficacy of elderly women. Journal of Physical Therapy Science, 25(7), 797–801. 10.1589/jpts.25.797 24259856 PMC3820389

